# Salt intake belief, knowledge, and behavior: a cross-sectional study of older rural Chinese adults

**DOI:** 10.1097/MD.0000000000004404

**Published:** 2016-08-07

**Authors:** Jing Zhang, Tao Wu, Hongling Chu, Xiangxian Feng, Jingpu Shi, Ruijuan Zhang, Yuhong Zhang, Jianxin Zhang, Nicole Li, Lijing Yan, Wenyi Niu, Yangfeng Wu

**Affiliations:** a The George Institute for Global Health at Peking University Health Science Center; b Peking University Clinical Research Institute, Beijing; c Changzhi Medical College, Shanxi; d First Hospital of China Medical University, Liaoning; e Xi’an Jiaotong University, Shaanxi; f Ningxia Medical University, Ningxia; g Hebei Province Center for Disease Prevention and Control, Hebei, China; h The George Institute for Global Health, University of Sydney, Australia; i Institute of Social Medicine and Health Service Management, Peking University School of Public Health, Beijing, China; j Department of Epidemiology and Biostatistics, Peking University School of Public Health, Beijing, China.

**Keywords:** behavior, belief, hypertension, knowledge, salt reduction

## Abstract

Excess sodium consumption is a major cause of high blood pressure and subsequent vascular disease. However, the factors driving people's salt intake behavior remains largely unknown. This study aims to assess the relationship of salt intake behaviors with knowledge and belief on salt and health among older adults in rural China.

A cross-sectional survey was conducted among 4693 older participants (men ≥50 and women ≥60 years old) randomly selected from 120 rural villages in 5 northern provinces in China. Healthy salt intake behavior was defined as either not eating pickled foods or not adding pickles/soy sauce/salt when food was not salty enough in prior 3 months.

There were 81% participants having healthy salt intake behavior. Healthy salt intake behavior was more common among women (*P* < 0.01) and was positively associated with age (*P* < 0.01) and poorer health status (*P* < 0.01), but negatively associated with years in school (*P* < 0.05). After adjusting for age, sex, years in school, and health status, participants who believed in the harm of high salt intake were more likely to have healthy salt intake behavior, compared with those who did not believe (Odds Ratio = 1.6, *P* < 0.001). Knowledge of salt intake was not significantly related to healthy salt intake behavior.

Our study demonstrated that belief in the harm of high salt intake rather than knowledge about salt and health was associated with healthy salt intake behavior, independent of age, sex, years in school, and health status. Future population salt reduction programs should place more emphasis on establishing health beliefs rather than only delivering salt-related knowledge.

Clinical trial registration number of the study is NCT01259700.

## 1 Introduction

Cardiovascular disease (CVD) is the leading cause of death in China, responsible for about 3.5 million (38%) deaths in 2008.^[1]^ Reflecting the major contribution of cerebrovascular disease to the vascular disease burden in China, high blood pressure is the leading modifiable risk factor for cardiovascular disease.^[2]^ Despite decades of remarkable advances in the treatment of hypertension, hypertension control remains an ongoing challenge for most countries. The problem is particularly marked in rural and northern China where sodium consumption, hypertension, and the incidence of stroke are all very high.^[3]^ There is a clear evidence from both observational studies^[4]^ and randomized trials^[5,6]^ that lower levels of salt consumption are associated with lower levels of blood pressure.^[6,7]^

However, the factors that drive salt intake behavior remains largely unknown and this significantly affects the development of interventions to reduce salt intake at a population level. It is natural to assume that behavior should be determined or affected by knowledge and by extension, understanding whether a person's salt intake behavior is related to their knowledge about salt and health would inform how salt reduction programs should be developed. Thus, this study aims to assess the relationship between salt intake behaviors and the perception of the harmfulness of high salt intake and knowledge about salt and health among older adults in rural northern China using data from the China Rural Health Initiative (CRHI). Studying the relationship between salt intake behavior and relevant beliefs and knowledge not only should be useful to rural Chinese population, but the knowledge gained from the study should be broadly generalizable to many other populations where there is a need for population salt reduction.

## 2 Methods

### 2.1 Study population

This study used data from the baseline survey of CRHI which was conducted in September 2010 in 120 rural villages across 5 provinces (Liaoning, Hebei, Shanxi, Shaanxi, and Ningxia). The details of the CRHI study design have been published elsewhere.^[8]^ The provinces were selected because of convenience and because both salt intake and cardiovascular disease burdens were high in their populations.^[9]^ Two counties were selected from each province based on interest and willingness to participate. Twelve townships were selected from each county through discussions with the leadership of the local county Bureau of Health. Within each township only one village was selected for the study.

All participants meeting the age requirement (men ≥50, women ≥60 years old) in the selected villages were eligible for participation and from those eligible approximately 40 people were selected randomly from each village (sex ratio 1:1) and invited to participate in the survey. Participants were excluded if they were mobility disabled, had difficulties for communication, had serious mental illness, hospitalized, or did not reside in the village for more than 4 months in the past year. Only one person in a family was invited to participate in order to avoid interindividual correlations. To do this, the first eligible person appeared on the roster of sampling would be invited first if more than one persons in the same family were drawn in the sample. In total, we recruited 5050 older adults from the 120 villages in 2 months.

The study was approved by the Peking University IRB (IRB00001052–10045). All participants provided signed informed consent form.

### 2.2 Survey methods

We trained local staff to conduct the CRHI baseline survey. Blood pressure measurements were taken with the participant in a sitting position after 5 minutes of rest. Two measurements were recorded at least 2 minutes apart using an automated electronic sphygmomanometer (Intellisense HEM – 7301 IT OMRON, Konan, Minato-ku, Tokyo, Japan). Heart rate, height, and weight were measured using standard procedures. The questionnaire included demographic information, lifestyle information, disease history, medication use, and medical care.

### 2.3 Defining and measuring the belief, knowledge, and behavior of salt taking

To understand participants’ belief of high salt intake on health, 1 simple closed question was asked about the effects of high salt intake on health (Belief). To define and measure knowledge about salt and health, participants were asked 2 closed questions, the effect of salt reduction on blood pressure (Knowledge 1, K1) and the salt intake daily upper limit recommended by the Chinese Nutrition Society (Knowledge 2, K2). To define and measure salt intake behavior, participants were asked 2 closed questions, the frequency of eating pickles (Behavior 1, B1) and their behavior when food did not taste salty enough (Behavior 2, B2). Table 1 shows the actual questions and closed answers and how we define “believed/not believed,” “known/unknown,” and “healthy/not healthy.”

**Table 1:**
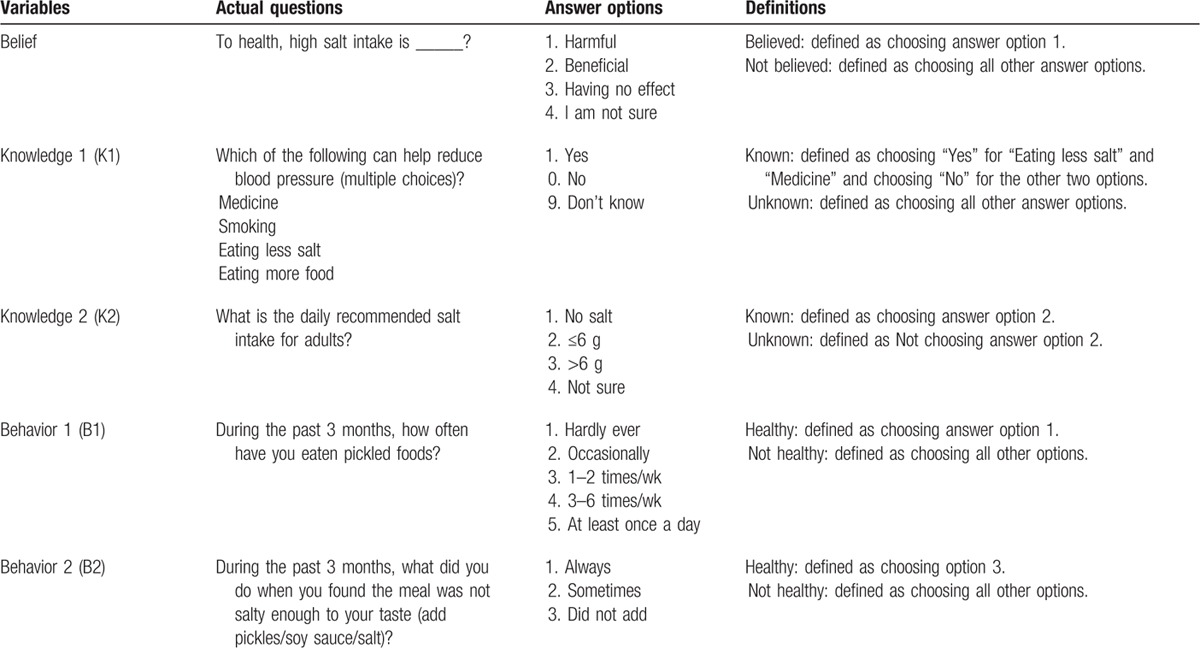
Illustration of the actual questions and definitions on knowledge and behavior about salt intake and health.

### 2.4 Statistical analysis

To be included in the analysis, we required the answers to all subquestions of Knowledge 1 question not be illogical to each other. Otherwise, the participants were excluded from the analysis. For example, for Knowledge 1, choosing “yes” to both “medicine” and “eating more food” was considered as illogical. We excluded 357 participants from the study for this reason. A total of 4693 participants remained for analysis. We performed χ^2^ test to justify the difference between populations included and excluded. There was no missing data in this study.

To summarize behavior measurements, we redefined healthy salt intake behavior as neither eating pickled foods nor adding pickles/soy sauce/salt into foods when food did not taste salty enough in the prior 3 months. We used logistic regression to analyze the association between healthy salt intake behavior and belief and knowledge, adjusting for potential confounders including age, sex, years in school, and health status. Because of the homogeneity of the participants being from the same village, we adjusted for cluster effects using generalized estimating equations (GEE).

To understand if the associations between belief, knowledge, and behavior were modified by participants’ health status, hypertension and cardiovascular disease in particular, we classified the study population into 4 health status groups: the “healthy” group if they had no hypertension or cardiovascular disease, “unaware hypertensive (HTN)” group if they had hypertension but was not aware of it, “aware hypertensive (HTN)” group if they had hypertension and were aware of it, and a “history of cardiovascular disease (CVD)” group including self-reported stroke and coronary heart disease. Hypertension was defined as having systolic blood pressure ≥140 mm Hg, diastolic ≥90 mm Hg, or/and having taken blood pressure lowering medications within the past 2 weeks.

## 3 Results

### 3.1 General characteristics

Table 2 shows the characteristics of study participants. Roughly half of the participants were men and half women with an age range from 50 to over 80. A large proportion (32%) of the study population was illiterate and only 17% had more than 9 years of education. About 20% had cardiovascular disease, another 49% had hypertension, and more than half were not aware of their health status. Less than a third (32%) were without cardiovascular disease or hypertension. In comparison with those excluded from the analysis, the participants included in the analysis were younger, included more men and were more healthy.

**Table 2:**
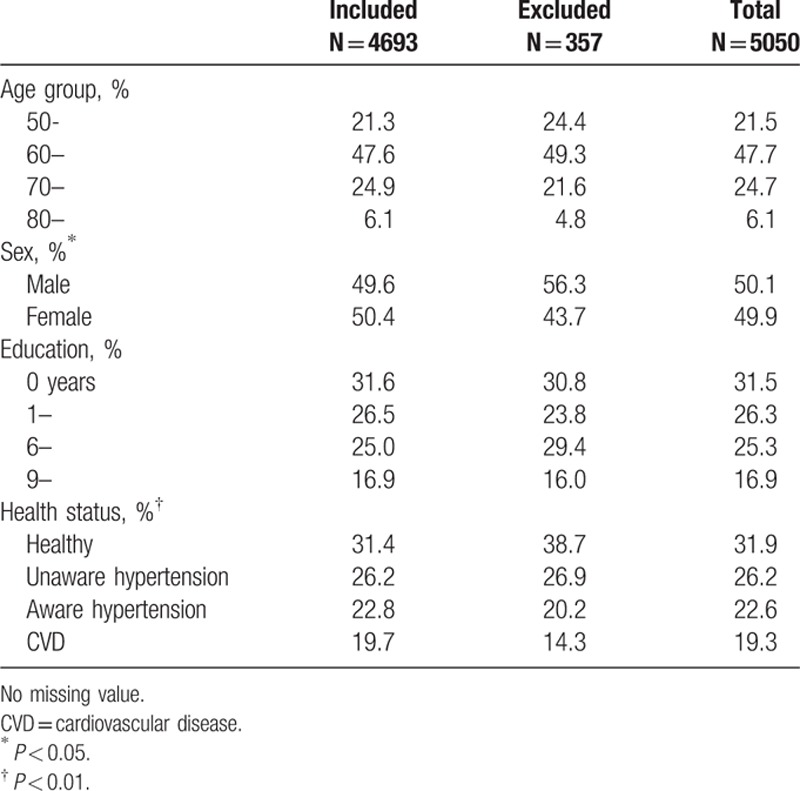
Characteristics of study population included and excluded.

### 3.2 Belief of the harmful effects of high salt intake

Overall, approximately 60% of study participants reported that they believed high salt intake would be harmful to their health. The belief about the harm of high salt intake was negatively associated with age, positively associated with years of school, and was slightly higher for women than men. From healthy group to unaware hypertension, to aware hypertension, further to the group with cardiovascular disease, the proportion with the belief significantly increased (Table 3).

**Table 3:**
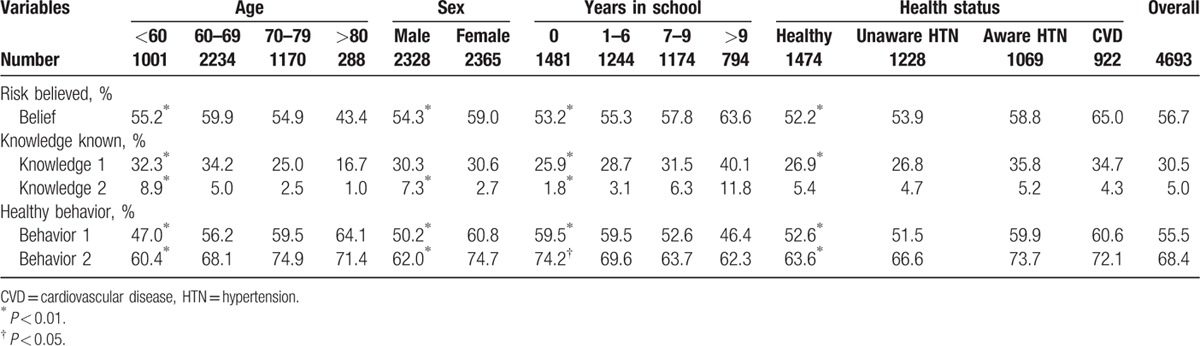
The percentage of participants having belief, knowledge, or healthy behavior by age, sex, years in school, and health status, among 4693 participants.

### 3.3 Knowledge about salt and health

In contrast to the fairly high proportion of participants who believed high salt intake is harmful to health, only about 30% knew that eating less salt can lower blood pressure, and only 5% knew the recommended daily salt intake upper limit. The older the study participant, the less they answered the questions correctly. The more years the study participants were educated, the more they answered the questions correctly. Across all health status groups from healthy group to the group with cardiovascular disease, the percentage of correct answers significantly got higher. However, the difference between men and women in knowledge was not consistent across the questions (Table 3).

### 3.4 Salt intake behaviors

Healthy salt intake behaviors were reported in 56%, 68%, and 81% of participants for B1, B2, and either B1 or B2, respectively. Unlike knowledge, the percentage of participants with healthy B1 and B2 behaviors increasingly associated with age and decreasingly associated with years of education, and was significantly lower in men than women. Consistent with knowledge, healthy behaviors positively associated with the health status across from the healthy group to the group with cardiovascular disease (Table 3).

### 3.5 The relationship between belief, knowledge, health status, and behavior

**Figure 1:**
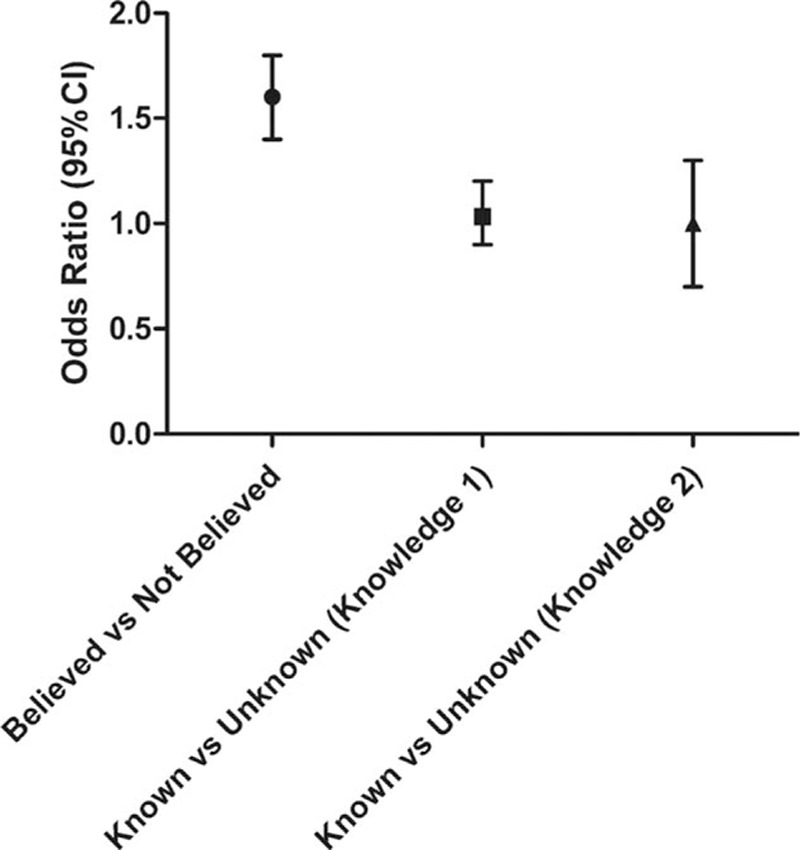
The relationship of healthy salt-taking behavior with the belief in harm of high salt intake and the knowledge of salt and health, after adjusting by sex, age, years of education, health status, and clustering effect.

To further clarify the relationships between belief, knowledge, health status, and behavior, we calculated the association of healthy salt intake behavior with belief, knowledge, and health status (Fig. 1 and Table 4). The results showed that belief was significantly associated with healthy salt intake behavior adjusting for health status, sex, age, and years of education. However, there was no significant association between healthy salt intake behaviors and knowledge about salt and health.

**Table 4:**
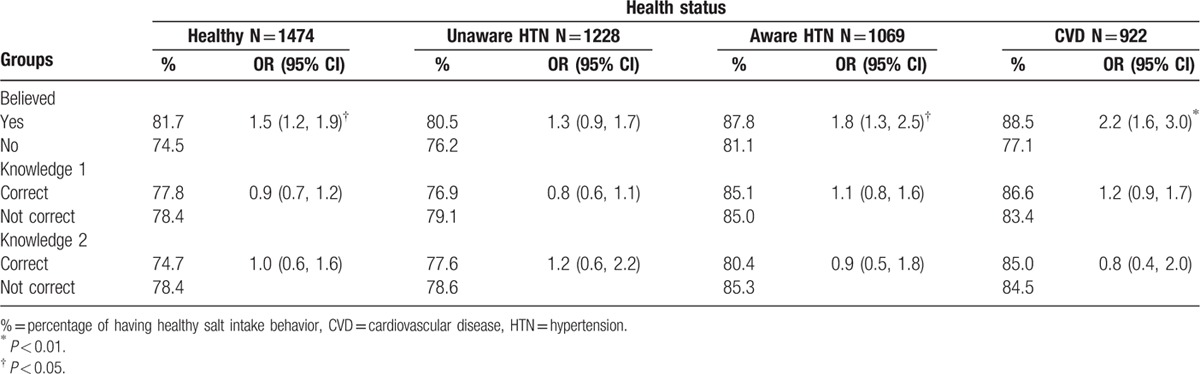
The relationship of healthy salt intake behavior with belief, knowledge, and health status, adjusted by sex, age, years in school, and clustering effect.

**Figure 2:**
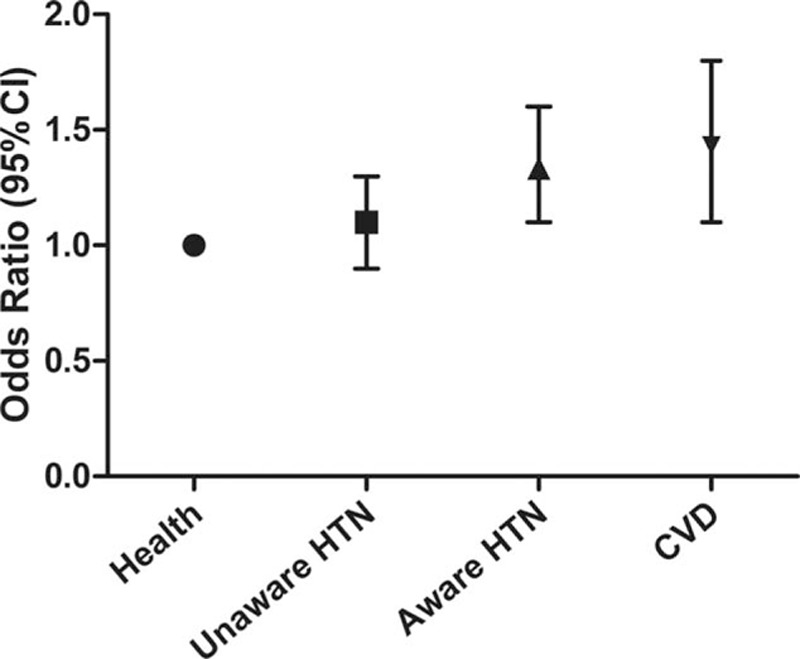
The relationship of healthy salt-taking behavior with health status, after adjusting by sex, age, years of education, belief, knowledge, and clustering effect.

Health status had a very strong impact on healthy salt intake behavior. Compared with apparently healthy participants, participants with CVD were most likely to have more healthy salt intake behaviors as well as those aware of their hypertension. However, those unaware of their hypertension were less likely to have more healthy salt intake behaviors, after adjusting for belief, knowledge, sex, age, and years of education (Fig. 2).

## 4 Discussion

Our study of a large random sample of older rural adults in northern China showed that healthy salt intake behavior was significantly associated with the simple belief in the harms of high salt intake rather than the knowledge of salt and health, and these associations were independent of age, sex, years of education, and health status. Our findings are well in line with Brenda Happell et al's^[10]^ study findings among patients with mental illness and support the Health Belief Model (HBM)^[11]^ which proposes that knowledge alone is insufficient to elicit behavior. This indicates that future population salt reduction programs should emphasize the importance of establishing a belief in the harms of high salt intake rather than simply delivering knowledge about salt and health.

Participants who completed this survey were older and less educated adults from rural China. Thus, it is no surprise that the proportion with knowledge about salt and health was low. In fact, only 5% of them knew the Chinese Nutrition Society's recommended daily amount of salt intake. However, a good proportion of them (nearly 60%) had the belief that high salt intake is harmful to health. And, 56% of them hardly ever ate salty food like pickles and nearly 70% would not add salt when they found food was not salty enough. These data further demonstrate that even among populations with little health knowledge and low education background, health belief could still be formed widely to influence health behavior.

The totality of a person's beliefs serves as the informational base that ultimately determines his attitudes, intentions, and behaviors.^[12]^ A person learns and forms a number of beliefs about an object on the basis of direct observation, or information received from outside sources or by ways of various inference processes. Clearly, the high proportion of the belief in the harm of high salt intake in our study participants was not from the participants’ direct observation or inference process that may be influenced by having knowledge on salt and health, because the proportion of participants with correct knowledge on salt and health was very low. Rather, it should be formed mainly on the basis of information received from outside sources. These outside information sources could include their families, neighbors, peers, community leaders that have an impact on the participant's belief formation. How to promote the formation of health beliefs among these disadvantaged populations remains a critical challege to the public health community. Our study finding that belief about the harms of high salt intake was significantly assciated with age, sex, years of education, and health status indicated that the HBM may be helpful and involving behavior scientists or psychologists in health education programs should be prioritized.

Our finding that healthy salt intake behavior was significantly higher among participants with hypertension and aware of it but not among those with hypertension but unaware of it was interesting and bears important value to public health practice. It suggests that increasing population awareness of hypertension could be an effective way for promoting healthy salt intake behavior resulting in better control of hypertension through less salt intake. Future salt reduction programs should consider including blood pressure measurement as a critical component.

Our study has some limitations. First, it was based on a cross-sectional design and hence no causal relationships can be drawn. Second, due to resource limitations we used a simple questionnaire to elicit participants’ belief, knowledge, and behavior focusing on salt and health to comply with the low education level of the study participants. This prevented us from conducting more sophiscated analysis, such as path analysis. Third, the study did not include people of a younger age or from urban areas. Generalization to other populations should be made cautiously.

In summary, our study found that a fairly large proportion of rural older Chinese had a health belief about salt and health, but in contrast, quite a small proportion had knowledge about salt and health. This belief was significantly associated with healthy salt intake behaviors. The study findings have suggested important new knowledge for designing and developing future population salt reduction programs and perhaps also other health education and health promotion programs.
